# Using past interglacial temperature maxima to explore transgressions in modern Maldivian coral and *Amphistegina* bleaching thresholds

**DOI:** 10.1038/s41598-021-89697-0

**Published:** 2021-05-13

**Authors:** Stephanie Stainbank, Dick Kroon, Erica S. de Leau, Silvia Spezzaferri

**Affiliations:** 1 Department of GeoSciences, University of Fribourg, Chemin du Musée 6, 1700 Fribourg, Switzerland; 2 School of GeoSciences, Grant Institute, University of Edinburgh, The King’s Buildings, James Hutton Road, Edinburgh, EH9 3FE United Kingdom

**Keywords:** Biogeochemistry, Climate sciences, Ecology, Ocean sciences

## Abstract

Tropical corals and *Amphistegina*, an example genus of symbiont-bearing larger benthic foraminifera, are presently living close to their thermal bleaching thresholds. As such, these essential reef-building organisms are vulnerable to the future prospect of more frequent sea surface temperature (SST) extremes. Exploring the earth’s paleo-climatic record, including interglacials warmer than present, may provide insights into future oceanographic conditions. We analyse foraminiferal shell geochemical compositions, from Recent surface sediments and Marine Isotope stage (MIS) 9e and MIS11c aged sediments, from the International Ocean Discovery Program Expedition 359 Site U1467 drilled in the Inner Sea of the Maldives. We illustrate through traditional (pooled) geochemical analysis (δ^18^O, Mg/Ca) that tropical temperatures were indeed marginally warmer during MIS9e and MIS11c in comparison to the modern ocean. Individual foraminiferal analysis (IFA) from the Recent (representing the last few hundred years) and MIS9e samples shows SSTs occasionally breached the coral bleaching threshold similarly to the modern-day. Significantly, the number of transgressions was four times higher during MIS11c, a recognised analogue for a warmer modern world. This new knowledge and novel IFA insight and application is invaluable given thermal stress is already obvious today with an increasing number of bleaching events over the last few decades.

## Introduction

**Figure 1:**
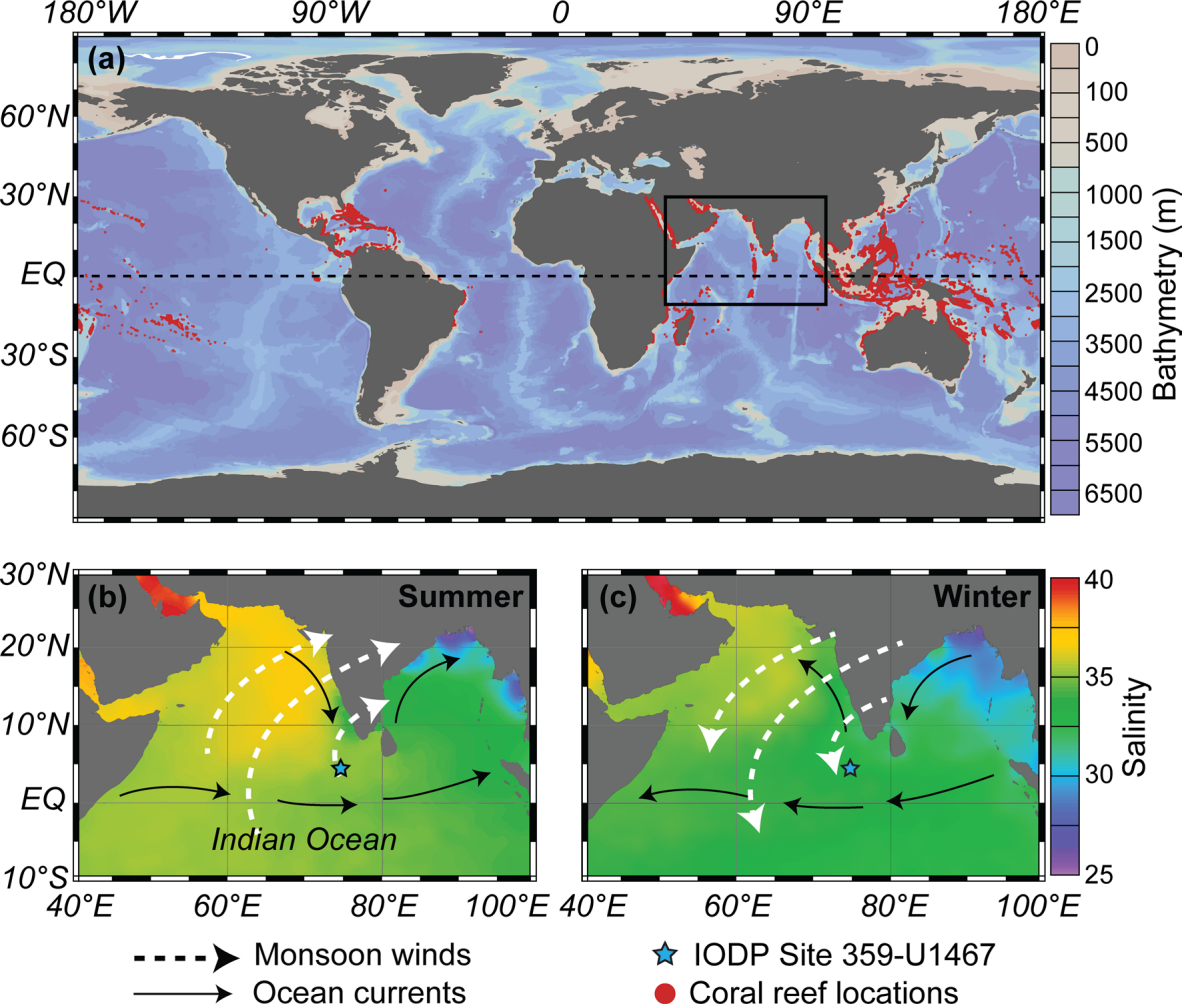
Global coral reef distribution and study area maps. **(a)** Global distribution of coral reefs (red dots) with the northern Indian Ocean salinity conditions shown for **(b)** summer and **(c)** winter. South Asian Monsoon wind (dashed arrows) and surface current (solid arrows) directions are indicated, with the location of the study site (IODP359-U1467^19^) marked by the blue star (coral reef locations from^61^ and all maps produced using Ocean data Viewer^62,63^).

It is undisputed that the future resilience, ecological functioning and ultimate survival of the world’s coral reefs is threatened^1,2^. These globally distributed tropical/subtropical ecosystems are biodiversity hotspots and fundamental socioeconomic components for innumerable countries, many of which already have extensive long-term monitoring and restoration programs in place^3^ (Fig. 1a). Due to the current sea surface temperature (SST) warming trends^4^, essential symbiont-bearing reef dwelling organisms are vulnerable. Both corals, the building blocks of reefs, and larger benthic foraminifera (~ > 500 µm) are important symbiont-bearing reef sediment contributors^5^. Considering their sensitivity to climatic perturbations, exemplified by the increase in duration and frequency of bleaching events over the last few decades, gaining insights into possible future scenarios is paramount. Looking within the earth’s paleo-climatic record could provide plausible future scenarios and extensive studies have identified multiple prospective analogues for our current Marine Isotope Stage 1 (MIS1) (e.g., MIS5e, 9e, 11c, 19^6,7^).

**Figure 2:**
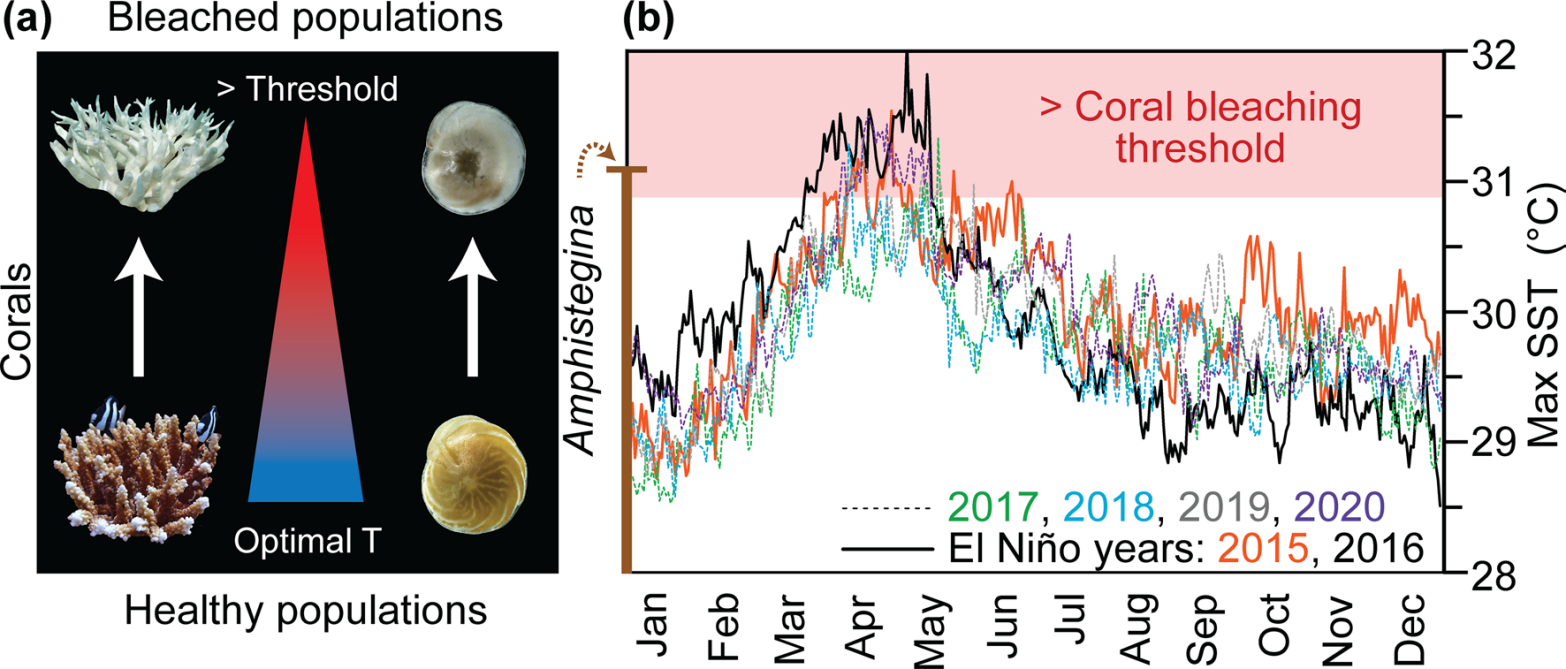
Bleaching thresholds. **(a)** Bleaching schematic for corals and the larger benthic foraminifera *Amphistegina* together with **(b)** maximum (Max) sea surface temperatures (SSTs) recorded in the Maldives for the years 2015–2020^10^. El Niño years (2015 and 2016) are represented by the solid lines with the years 2017–2020 represented by dashed lines. Temperatures > the coral bleaching threshold for the Maldivian coral reefs (~ 30.9 °C^10,25^) are indicated in red shading. δ^18^O_c_ temperature estimates derived from living (Rose Bengal stained) *Amphistegina lessonii*, from the Maldives, are also shown (vertical brown line).

The reef-building Scleractinian corals, marine invertebrates that secrete calcium carbonate (aragonite) skeletons, have a mutualistic relationship with microalgae symbionts (zooxanthellae) which are essential to their survival^8,9^ (Fig. 2). Similarly, *Amphistegina*, a larger benthic foraminifera (unicellular protist: ~ 500 µm-3 mm) that secretes a calcium carbonate (calcite) shell (test), has a mutualistic relationship with its symbionts (diatoms)^5^ (Fig. 2). Under high SSTs, these organisms become stressed and are susceptible to bleaching, which implies either the expulsion or digestion of their symbionts leaving them with a characteristic white appearance^8,9^ (Fig. 2). Over prolonged and/or more frequent warm events (e.g., El Niño events), the potential for corals and *Amphistegina* to recover diminishes, which can lead to high mortality events and even the ultimate demise of entire reefs, as seen globally in the El Niño related mass-bleaching’s of 1998, 2010 and 2016. As such, assessing transgressions in bleaching thresholds is important to aid in our understanding of these fragile biologically and economically significant ecosystems. Coral bleaching thresholds are defined by^10^ as SSTs 1 °C higher than the highest monthly mean, placing the regional Maldives coral bleaching threshold at ~ 30.90 °C, with^11,12^ denoting a similar bleaching threshold for *Amphistegina* at 31 °C (Fig. 2).

The benthic foraminiferal genus *Amphistegina* lives on reefs at depths of ≤ 50 m and has a similar bleaching and thermal limit to corals, as well as a long (relative to other smaller foraminifera) life cycle (up to 12 months). On the contrary, within the top ~ 100 m of the pelagic realm, live the smaller (~ < 500 µm) symbiont-bearing, omnivorous, shallow-dwelling planktonic foraminiferal species (e.g., *Globigerinoides ruber* (white) and *Trilobatus sacculifer*) which can withstand greater temperature (14–32 °C) as well as salinity (22–49 PSU) extremes^13^ and have a life cycle of usually 2–4 weeks. Importantly, planktonic foraminiferal shells are readily preserved within the sedimentary environment of the deeper pelagic ocean, adjacent to the shallow and often turbulent coral reef settings, which provides a more stable location whereby paleoenvironmental changes are potentially continuously recorded at high temporal resolution. Through geochemical analysis of the fossil shells of these planktonic species, with a predisposition to have lived within the warmest upper reaches of the water column due to the light dependency of their symbionts, we can extract the variability in temperature extrema for the tropical surface ocean, over temporal scales beyond that of modern-day instrumental records. In addition, due to their short life cycles, these planktonic species provide the potential to capture the temperature extremes experienced during brief periodic warm events, which would otherwise not be captured by studying larger benthic foraminifera or corals. As such, they provide the possibility to test how far beyond and how regularly bleaching thresholds, as observed in the modern, were exceeded during temperature maxima of past warmer interglacials.

**Figure 3:**
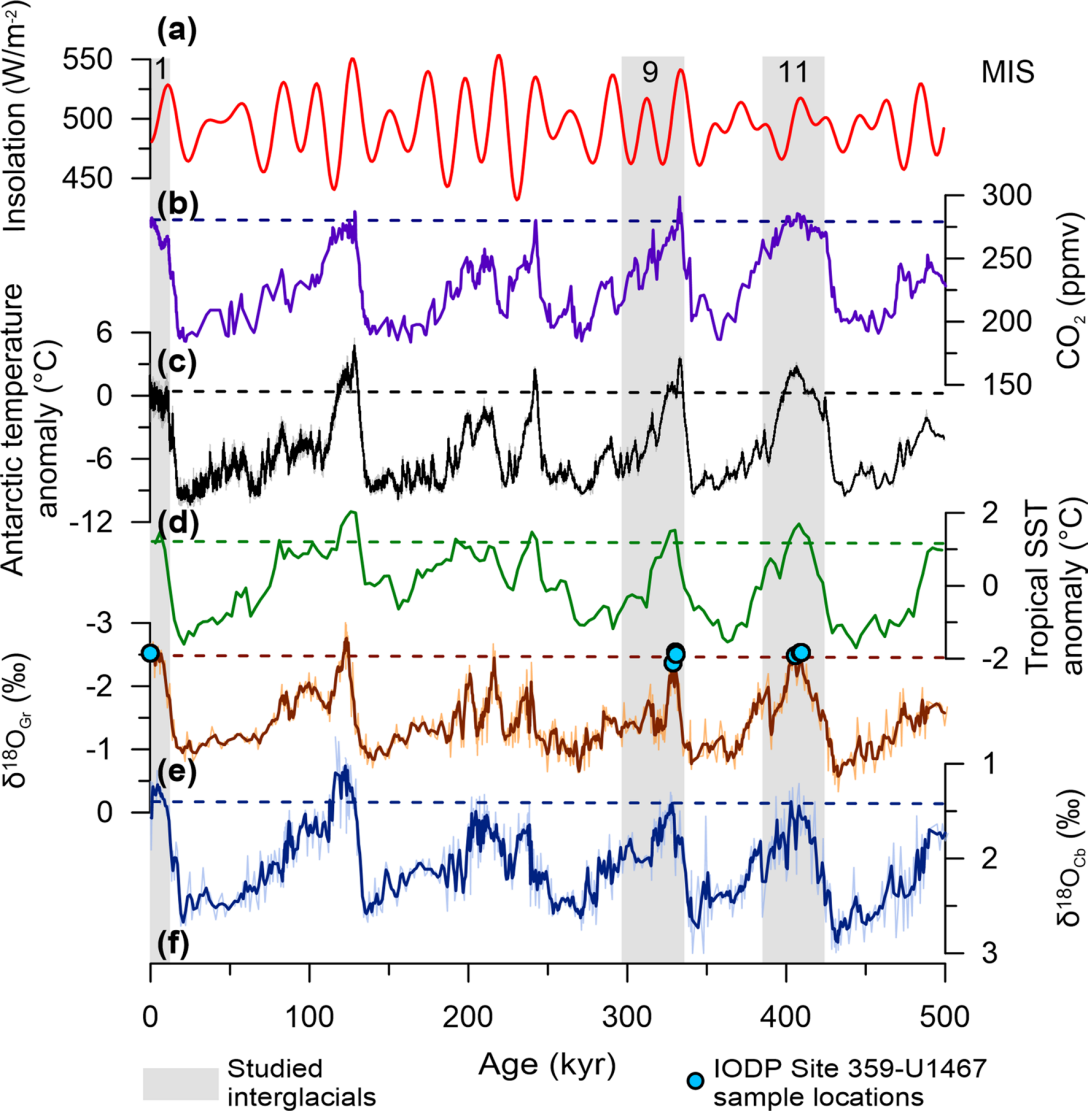
Long-term climatic records for the last 500 kyr. **(a)** Insolation patterns for 65°N^64^; **(b)** Compilation of atmospheric CO_2_ levels^14^; **(c)** Antarctic temperature anomalies relative to mean temperature over the last millennium^16,66^; **(d)** Tropical U^K^’_37_ SST anomalies relative to their modern mean^26,67^; **(e)** Maldives (IODP Site 359-U1467) planktonic *G. ruber* δ^18^O_c_ record^45,65^ and **(f)** Maldives (IODP Site 359-U1467) epibenthic *C. mabahethi* δ^18^O_c_ record^45,65^. Dashed lines show the modern-day levels with the exact location of IODP Site 359-U1467 samples used in this study shown (blue circles). MIS = marine isotope stage with MIS1, MIS9 and MIS11 highlighted in grey shading, SST = sea surface temperature.

While there might not be an ideal period of time, which inherently encapsulates our anthropogenically enhanced rapid warming trend, intervals with similar insolation patterns (e.g., MIS11c), and/or greater atmospheric CO_2_ levels (e.g., MIS9e) could be sufficient to observe variability in tropical SSTs in a world warmer than present. Within the last 500 kyr of earth’s history, MIS11 fits this profile, and is considered the closest warm period analogue to the Holocene^6,7,14^, due to its weak precessional forcing and similar insolation trends as well as its climatic extreme (MIS11c) being reportedly 0.21- 5 °C warmer^15–18^ (Fig. 3). The MIS9 interglacial, while possessing different astronomical forcings, its climatic extreme (MIS9e) is the second warmest over this period with the highest atmospheric CO_2_ levels (~ 300 ppm) (Fig. 3).

Within this context, we have studied samples from the tropical Maldivian archipelago from MIS9e and MIS11c and compared them with the Recent environment (= mudline sample representing the last few hundred years, see “Methods” section) (exact sample locations are shown on Fig. 3e). Cores were retrieved from a depth of 487 m during the International Ocean Discovery Program (IODP) Expedition 359 from the Inner Sea of the Maldives archipelago; a low latitude, oligotrophic region (3.2028° N, 73.2207° E) within the Indian Ocean^19^. The Maldives has extensive coral reef ecosystems, which includes both corals and larger benthic foraminiferal shoals, all of which were adversely impacted by all three of the recent mass-bleaching events.

Individual foraminiferal analysis (IFA) as well as traditional whole-shell (whole-test) analyses, based on pooled specimens, were conducted to measure the geochemical compositions (δ^18^O_c_ and Mg/Ca) recorded in the calcitic shells of the two most commonly utilised (for surface ocean paleoreconstructions) shallow-dwelling, symbiont-bearing planktonic foraminiferal species: *G. ruber* (white) and *T. sacculifer* (with sac-like final chamber). We use these geochemical measurements, which have been extensively applied in paleoceanographic studies^20–22^, as proxies for SST and δ^18^O of seawater (δ^18^O_sw_).

While traditional whole-shell measurements (based on 2–100 pooled specimens) provide indications of mean hydrographic conditions, IFA (based on the measurement of single specimens) has been previously used to study present and past seasonality and hydrographic variability^20,23,24^. We apply IFA to assess the temperature extrema, for Recent, MIS9e and MIS11c samples in relation to the modern-day coral bleaching-threshold (~ 30.90 °C) for the Maldives region^10,25^. The rational to apply the IFA approach on planktonic foraminiferal species is based on their short life cycle (2–4 weeks) which implies that individual specimens live within or straddle the seasonal cycles. Thus, by measuring multiple specimens, the warmest (often short-lived) intervals can be captured and provide a window into how often and by what magnitude, the modern-day bleaching threshold was exceeded during the warmer MIS9e and MIS11c. This knowledge is essential to enhance predictability and understanding of the behaviour of bleaching susceptible organisms within coral reef ecosystems (e.g., corals and *Amphistegina*). Moreover, this insight is crucial given the future prospect of more frequent El Niño events, which will place these organisms at risk and under increasing strain, particularly in the case of the Maldives if SSTs continuously reach and surpass the local bleaching threshold of ~ 30.90 °C (Fig. 2b).

## Results

### Present and past SST extrema

Over the last 35 years, the maximum SST instrumentally recorded in the Maldives was ~ 32.98 °C, significantly beyond both the coral and *Amphistegina* bleaching thresholds, with the highest SSTs consistently recorded during El Niño events^10^ (Fig. 2b). The studied *Amphistegina lessonii* specimens, collected live (Rose Bengal stained) from surface sediments during the 2015 El Niño, record a maximum temperature of 31.11 °C (mean = 29.65 ± 1.43 °C) at the brink of its thermal tolerance (31 °C^11,12^). These temperature calculations, derived from δ^18^O_c_ signatures preserved in the calcitic shells, are comparable with in situ instrumentally recorded SST measurements (Fig. 2b). This attests to the reliability and applicability of foraminiferal geochemical proxies, which can rely on either δ^18^O_c_ or Mg/Ca measurements, to calculate temperature estimates.

**Figure 4:**
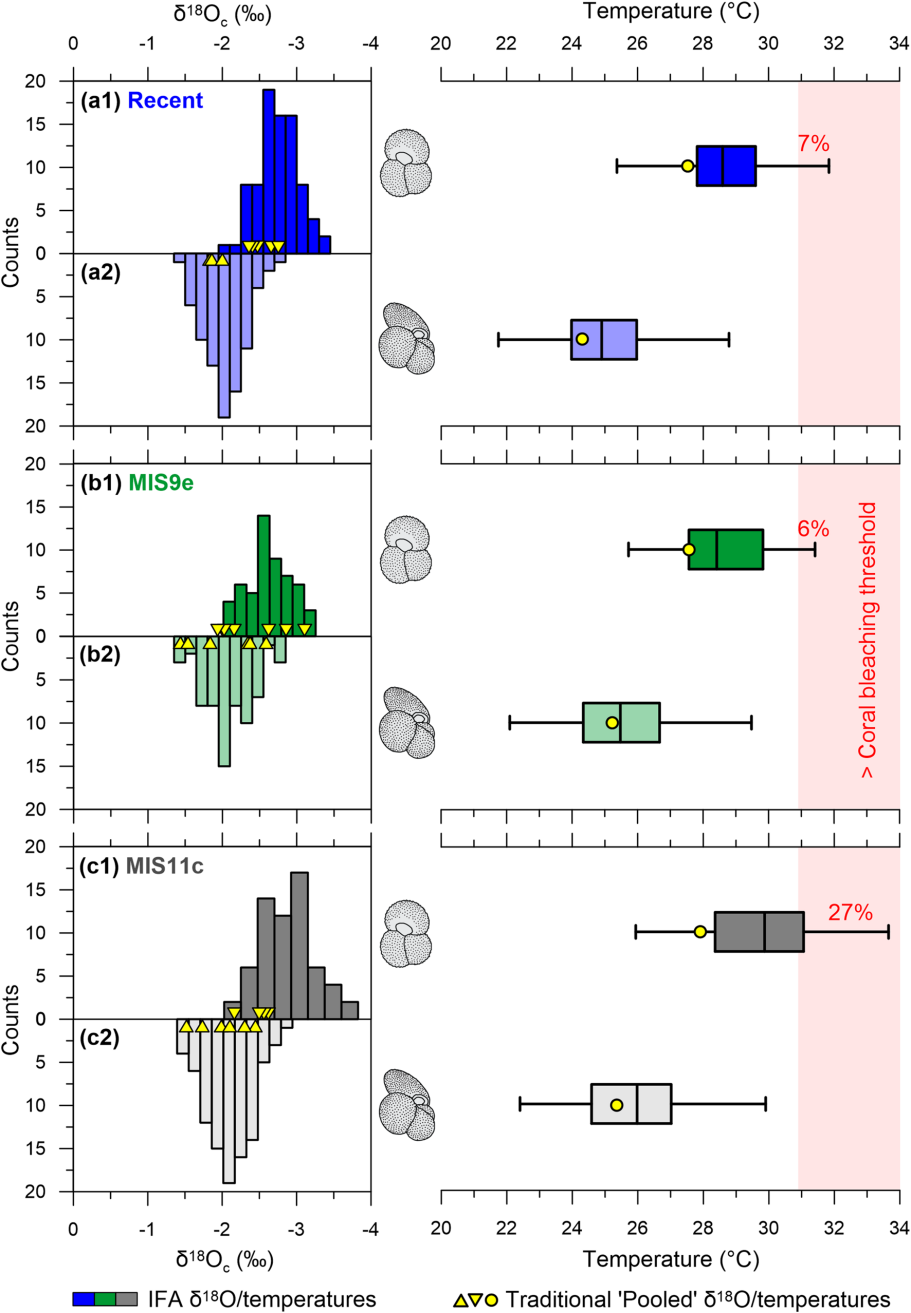
Spread in δ^18^O_c_ individual foraminiferal analysis (IFA) data and seawater temperature estimates. Histograms of the δ^18^O_c_ IFA data spread (counts = number of measurements) and the corresponding temperatures (box and whisker plots) for **(a)** the Recent (blue), **(b)** MIS9e (green) and **(c)** MIS11c (grey) for both **(1)** *G. ruber* (w) and **(2)** *T. sacculifer* (w/s). Traditional δ^18^O_c_ values (based on pooled specimens) are shown (yellow triangles) for each sample together with the mean δ^18^O derived temperature (based on pooled specimens) (yellow circles). Temperatures > the Maldives coral bleaching threshold (~ 30.9 °C^10^) are shown in red shading. Numbers indicate the percentage of the IFA temperature estimates, from each dataset, > this bleaching threshold. Note: MIS9e and MIS11c represent the pooled data from three samples.

The long-term Antarctic dataset of^16^ shows a maximum higher latitude SST deviation of + 3.75 °C and + 3.15 °C at the peaks of MIS9e and MIS11c, in comparison to modern SSTs (Fig. 3). In contrast, the tropical alkenone SST stack of^26^ report a ~ 1.52 °C and ~ 1.70 °C increase, from modern SSTs, for each time interval with the MIS11 modelling study of^15^ showing only a ~ 0.25–0.50 °C increase across the tropics. During extreme Pleistocene climatic excursions (e.g., during the warmer MIS9 and MIS11 interglacials) the tropics are anticipated to be more climatically stable in comparison to the higher latitudes, due to several feedback and/or thermostatic regulation mechanisms^15,18,27^. This dampened expression is similarly recorded by our traditional whole-shell (pooled) *G. ruber* (w) and *T. sacculifer* (w/s) geochemistry data (Mg/Ca), which record a mean temperature increase of only ~ 0.45 °C and 0.66 °C for MIS9e and MIS 11c, respectively in comparison to the Recent (Fig. 4). Yet, these mean tropical seawater temperatures, recorded by *G. ruber* (w) (x̄ Recent = 27.56 ± 0.70 °C; MIS9e = 27.59 °C ± 2.08 °C; MIS11c = 27.82 ± 0.64 °C) and *T. sacculifer* (w/s) (x̄ Recent = 24.33 ± 0.45 °C; MIS9e = 25.21 ± 2.40 °C; MIS11c = 25.38 ± 1.39 °C), do not necessarily reflect the true upper temperature limits of the SML within which these species lived, as their living depths are governed by ecological preferences (Fig. 4). These symbiont-bearing and omnivorous species have a reported affinity for the deep chlorophyll maximum (DCM) with *G. ruber* (w) having a thermal preference of ~ 27 °C and *T. sacculifer* (w/s) known to live slightly deeper in cooler waters^28–30^. However, when compared with the widespread IFA δ^18^O_c_ data, it is evident that periodic extreme warm events are unlikely to be captured using the traditional whole-shell (pooled) geochemical methods (Fig. 4).

The Recent IFA *G. ruber* (w) temperature spread has an upper limit of 31.84 °C, which is similar to the maximum SST recorded in the Maldives over the last six years (= 32.98 °C), with 7% of the data above the modern-day coral bleaching threshold (30.9 °C). Comparable excursions were observed during MIS9e and MIS11c, with upper IFA temperature limits of 31.41 °C and 33.66 °C, respectively. Furthermore, these IFA upper temperature limits are substantially warmer than the recorded means from the traditional pooled measurements (Fig. 4). More revealing than their upper limits, 6% and 27% of the MIS9e and MIS11c *G. ruber* (w) IFA temperature estimates are beyond the modern Maldivian coral bleaching threshold (30.9 °C). Overall, the *T. sacculifer* (w/s) data records cooler temperatures than *G. ruber* (w), yet still illustrates an increase in the upper temperature extremes for both MIS9e and MIS11c in comparison to the Recent dataset. While the MIS9e datasets are not statistically different from the Recent, there is a significant difference with both the MIS11c *G. ruber* (w) (t = − 4.1471, p = 0.000) and *T. sacculifer* (w/s) (t = − 3.3793, p = 0.001) datasets.

## Discussion

### Future insights and prospects

Increased thermal stress and its associated impacts, on tropical marine ecosystems, is a primary concern for the 21st century. Over the last 100 years, SSTs have risen by more than 1 °C within these ecologically sensitive areas^4^. Alarmingly, the rate of ocean warming is still expected to increase three to four-fold by the end of this century^4^. A warming climate will further promote more frequent as well as more severe El Niño events^31^, continuously pushing SSTs above coral and *Amphistegina* bleaching thresholds. These climatic projections may have disastrous effects on our coral reef ecosystems that are home to some of the highest biodiversity on earth and are extremely sensitive to thermal perturbations. Moreover, it is estimated that coral reefs contribute up to $37.8 billion a year to the world’s economy^32^. Thus aside from a biological viewpoint, protecting these ecosystems is vital for the economic survival of many countries, including the Maldives, who are reliant on them for both “on-reef” and “reef-adjacent” tourism.

Coral reefs and their associated benthic foraminiferal shoals are intrinsic ecological communities, made up of a wide array of fauna and flora, many of which are already living close-to or at their thermal limits^33^. Corals, sedentary marine animals and the building blocks within these tropical realms, and *Amphistegina*, an example genus of larger benthic foraminifera, are particularly vulnerable due to their reliance on symbionts and as such shallow habitats. Furthermore, due to the slow growth rates of corals (0.3–10 cm/year^34^) protracted recovery times are evident following mass-bleaching events, which could prove detrimental, not only for corals but for tropical shallow water ecosystems as a whole. Thus, a future increase in frequency, magnitude and more importantly duration of periodic warm periods is of concern as it impedes the ability of both corals and *Amphistegina* to recover and this threat further supports the value of using past analogues to gain insight into possible future conditions.

Our novel application of the IFA technique highlights its potential in assessing temperature extrema. While the traditional (pooled) geochemical approach is favourable when constructing long-term records, as it ensures the data is less biased by outliers and presents a more representative mean for each time point, the variability in the data is lost. This demonstrates the applicability of the IFA technique if transgressions in the upper temperature limits are of interest, which is presently the primary threat for coral reef ecosystems. While our Recent and MIS9e datasets are similar and not statistically different, MIS11c is notable, clearly showing warmer SSTs. Considering it is the closest warm period analogue to the Holocene, within the last 500 kyr of earth’s history, it is a potential avenue to gain insights into future scenarios.

Our study on Maldivian, shallow-dwelling symbiont-bearing planktonic foraminifera confirms previous studies showing higher mean seawater temperatures in MIS11c (+ ~ 0.66 °C) in comparison to the Recent. In addition to the mean populations sitting closer to the current coral bleaching threshold during MIS 11c, the IFA datasets specify a shift towards higher temperature extremes. Moreover, the MIS11c *G. ruber* (w) IFA dataset is skewed towards the lowest δ^18^O_c_/highest temperature values, implying an increase in periodic warming events (Fig. 4). Here we recognise the potential for IFA δ^18^O_c_ datasets, to provide supplementary information from the traditional (pooled) foraminiferal geochemical analyses. As opposed to pooled measurements, which provide a mean signal of the measured population, it is possible to extract temperature extrema from IFA data and allows us to gain insights in the relative frequency and magnitude of periodic warm events. Based on the reality that current anthropogenic warming is much faster than seen during MIS11, more extreme as well as more frequent El Niño events are anticipated in the future^31^. Furthermore, we expect that the number of transgressions in the coral and *Amphistegina* bleaching thresholds will increase beyond that currently observed in both the Recent, MIS9e and MIS11c shallow water planktonic foraminiferal assemblages. As such, insight gained through IFA studies is invaluable given that thermal stress is already obvious today with an increasing number of global bleaching events, associated with high coral and *Amphistegina* mortality, over the last decades placing the world’s coral reef ecosystems under ever increasing strain.

## Methods

### Study site and target foraminiferal species

The Maldivian archipelago is a partially drowned carbonate platform within the central, equatorial Indian Ocean. It consists of two rows of north–south orientated atolls, which encompass an Inner Sea. The lowermost neritic carbonate unit sits upon volcanic bedrock and has been dated back to the Eocene^19^ with continuous drift deposition, within the Inner Sea, starting ~ 12.9 Ma at the establishment of the modern South Asian Monsoon (SAM)^35,36^. This seasonally reversing, major climatic system has an impact on both the regional precipitation patterns as well as physiochemical oceanographic properties (Fig. 1). The summer southwest SAM brings warm, wet conditions to the Indian subcontinent, as well as higher saline surface waters from the Arabian Sea into the Maldives region. In comparison, the winter northeast SAM results in cool, dry continental conditions and transports lower salinity water from the Bay of Bengal into the central, equatorial Indian Ocean. As a result, the Maldives seasonal salinity depth profiles can vary significantly, yet due to its tropical location the seasonal sea water temperatures are relatively stable.

Three symbiont-bearing foraminiferal species are used in this study: *Amphistegina lessonii*, *Globigerinoides ruber* (white) and *Trilobatus sacculifer* (with sac-like final chamber):

*Amphistegina lessonii* is a larger benthic, symbiont-bearing (diatoms) foraminiferal species. It has a shallow depth range (0–50 m)^37–39^ and is globally abundant in tropical coral reef, benthic foraminiferal shoal and general carbonate shelf settings^40^. Similarly to corals, amphisteginids have been shown to bleach under high temperatures/high irradiance levels with the new development of the *Amphistegina* Bleaching Index (ABI) as an indicator of photo-inhibitory stress in coral reef settings^41,42^. From ~ 30 °C this species starts showing signs of thermal stress, with bleaching and mortality reported for temperatures > 31 °C^11,12^.

*Globigerinoides ruber* (w) hosts dinoflagellate endosymbionts and is the most common planktonic foraminiferal species in tropical-subtropical waters^13^ state that while *G. ruber* (w) is generally considered one of the shallowest-dwelling species, its depth distribution does vary in relation to regional ecological conditions. It has a particular relation to the nutricline depth in less turbid, oligotrophic conditions^43^ which has been confirmed for the Maldives^28^. It is omnivorous, however in comparison to other omnivorous, symbiont-bearing species, it has demonstrated an elevated adaptation for consuming phytoplankton protein over zooplankton protein^13^. From culture experiments, it has a broad temperature (14–31 °C) and salinity (22–49 PSU) tolerance, and has been reported as the most tolerant species to low sea surface salinity (SSS)^13^. This species occurs year-round and has a fortnightly reproduction^13^.

*Trilobatus sacculifer* is a planktonic foraminiferal species abundant in tropical-subtropical surface waters and as such is extensively used in paleo-reconstructions. It hosts dinoflagellate endosymbionts yet is omnivorous, feeding predominantly on calanoid copepods^13^. It is a euryhaline species, with a broad salinity (24–47 PSU) and temperature (14–32 °C) tolerance. Similarly to *G. ruber* (w), this species occurs year-round and has a monthly reproduction on a synodic lunar cycle^13^. While a shallow dwelling species, it is generally reported to live slightly deeper in the water column, in comparison to *G. ruber* (w)^28,30,44^.

### Sampling

All planktonic foraminiferal specimens (*G. ruber* (w) and *T. sacculifer* (w/s)) for the geochemical analysis (δ^18^O_**c**_ and Mg/Ca) originate from the International Ocean Discovery Program (IODP) Expedition 359, Site U1467 (4° 51.0274′ N, 73° 17.0223′ E) drilled in 2015 within the Inner Sea of the Maldivian archipelago at a water depth of 487 m^19^. The age model for these samples was adopted from a previous study^45^ which is based on the correlation of their long-term (0–1800 kyr) Site 359-U1467 *C. mabahethi* and *G. ruber* (w) δ^18^O_**c**_ records to the stacked reference curve of^46^. Recent surface sediment samples (mudline A and B: representing the sample from the sediment/water interface), as well as three samples from the peak of MIS9e (U1467C, 2H6, 0–1 cm; U1467C, 2H6, 15–16 cm; U1467C, 2H6, 18–19 cm) and MIS11c (U1467B, 3H2, 147–148 cm; U1467B, 3H3, 9–10 cm; U1467B, 3H3, 12–13 cm) were analysed^19,28^ (sample locations are shown on Fig. 3). The mudline is identified as Recent, likely representing the last few hundred years, based on the presence of Rose Bengal (1 g/L) stained ostracods and benthic foraminifera. The study by^45^ has verified that diagenetic influences, within this shallow, carbonate environment, are not a concern for foraminiferal geochemical compositions over the investigated time-interval (MIS1-11).

Rose Bengal stained *A. lessonii* specimens were obtained from modern surface rubble samples collected by hand, at 10 m water depth, during the 2015 International Union for Conservation of Nature (IUCN) REGENERATE cruise^47^ (Supplementary Table 6). Samples were collected from the reefs of two islands, Maayafushi and Rasdhoo, both located within the central part of the Maldivian archipelago. As the foraminifera shells were stained pink, it implies they were living at the time of collection. These specimens were used for stable isotopic analysis and their reconstructed temperatures represent modern (a cumulative signal encompassing their lifespan of four to twelve months^48^) conditions (Supplementary Tables 5–6). A full explanation of the Rose Bengal protein stain for foraminifera is detailed in^49^.

### δ^18^O_c_ stable isotopic analysis

All samples were initially washed using a 32 μm sieve to remove the finer clay and silt fractions. Subsequently, they were air dried and sieved into discrete sizes for foraminiferal picking. To ensure enough calcite for the measurements, all specimens for Individual Foraminifera Analysis (IFA) for both *G. ruber* (w) and *T. sacculifer* (w/s) (n = 632) were picked from the 355–400 μm size fraction. In addition, traditional whole-shell (pooled) measurements for *G. ruber* (w) (n = 24) were conducted on specimens from the 212–400 μm fraction (2–5 pooled specimens). *Trilobatus sacculifer* (w/s) traditional whole-shell analysis (n = 21) was measured on specimens (2 pooled specimens) from the 300–355 μm fraction. The majority of these pooled measurements are obtained from^28,45,50,51^ (Supplementary Table 1). *Amphistegina lessonii* measurements were run on single specimens > 250 μm in size. Prior to stable isotopic analysis, all shells were briefly cleaned (1–2 s) by ultrasonication in Milli-Q water to remove any adhering particles. All stable isotopic measurements were conducted at the School of GeoSciences at the University of Edinburgh on a Thermo Electron Delta + Advantage mass spectrometer integrated with a Kiel carbonate III automated extraction line. Samples were reacted with 100% phosphoric acid (H_3_PO_4_) at 90 °C for 15 min, with the evolved CO_2_ gas collected in a liquid nitrogen coldfinger and analysed compared to a reference gas. All samples are corrected using an internal laboratory standard and expressed as parts per mil (‰) relative to Vienna Pee Dee Belemnite (VPDB). Replicate measurements of the standards give the instrument an analytical precision (1σ) of ~ 0.05 ‰ for δ^18^O and δ^13^C.

### Mg/Ca analysis

The Mg/Ca data is obtained from^28,45,50,51^ (Supplementary Table 1). Each *G. rub*er (w) Mg/Ca analysis (n = 17; 212–250 μm in size) was conducted on 30 pooled specimens by inductively coupled plasma optical emission spectrometry (ICP-OES) on a Thermoscientific iCap 6300 (dual viewing) at the Institute of Geosciences of the Goethe-University of Frankfurt. All samples were initially cleaned (1–2 s) by ultrasonication in Milli-Q water and then the standard oxidative cleaning protocol of^52^ followed to prevent clay mineral contamination. The final centrifuged sample solution was diluted with an yttrium solution (1 mg/l) prior to measurement to allow for the correction of matrix effects. In addition, before each analysis five calibration solutions were measured to allow for intensity ratio calibrations. All element/Ca measurements were standardized using an internal consistency standard (ECRM 752–1, 3.761 mmol/mol Mg/Ca). Furthermore, the elements Al, Fe, and Mn were screened and blanks periodically run to monitor for further signs of contamination during the analyses.

### Establishment of present and past seawater temperatures

Prior to temperature calculations, we test the IFA distributions for normality using the Shapiro‐Wilk test and the Fisher–Pearson coefficient of skewness with bootstrap confidence intervals, to define the skewness of the datasets^53^ (Supplementary Table 3). The Recent *G. ruber* (w) and *T. sacculifer* (w/s) and MIS11c *T. sacculifer* are normally distributed. In the case of both MIS9e datasets and the MIS11c *G. ruber* population, the null hypothesis that the data are normally distributed (p ≤ 0.05) is rejected (Supplementary Table 3). Considering bioturbation within the sediment record is a possibility, we use two methods to identify and remove outliers in the IFA datasets. Firstly, the inter-quartile range (IQR) is used for each δ^18^O_c_ dataset, which defines a measurement as an outlier if it falls outside the range [Q1 − 1.5 (Q3 − Q1), Q3 + 1.5 (Q3 − Q1)], with IQR = Q3 − Q1 and Q3 and Q1 representing the third and first quartile of the dataset^20^. But if there is considerable reworking, the IQR method would not necessarily identify reworked glacial measurements (highest δ^18^O_c_ values) within the interglacial samples. As such, the Recent IFA datasets, which are both normally distributed, are used to further set a rudimentary cut-off point for the highest δ^18^O_c_ (= lowest temperatures) value to expect during past interglacial minima periods for both *G. ruber* (w) and *T. sacculifer* (w/s) (this is discussed further in the Supplementary Materials, Supplementary Figs. 1–3).

There are innumerable analytical techniques (e.g., traditional mass spectrometry, secondary-ion mass spectrometry, laser ablation inductively coupled plasma mass spectrometry), proxies (Mg/Ca, δ^18^O, clumped isotopes, TEX86, U^k^’_37_) as well as target medians (e.g., calcitic shells of foraminifera, aragonitic coral skeletons, ice, lipids, alkenones) which are used in marine paleo-temperature reconstructions. Furthermore, different methods exist in the literature to calculate temperature estimates using both planktonic foraminiferal δ^18^O_c_ and Mg/Ca measurements with innumerable species-specific δ^18^O-temperature and Mg/Ca-temperature equations reported^20,23,30,54–56^. Moreover, due to the exponential nature of the Mg/Ca-temperature equations, if inappropriately applied, offsets in the upper temperature range are exacerbated. Additional considerations are species-specific offsets and differential geochemical compositions within the shell (e.g., high versus low Mg banding, gametogenic calcite). *Trilobatus sacculifer* gametogenic calcite has been reported to be significantly enriched in Mg in comparison to the rest of the shell^57^. As *T. sacculifer* specimens selected for use in this study underwent reproduction, indicated by the presence of a sac-like final chamber^58^, we can expect their Mg/Ca ratios to be biased. As such, to avoid overestimates we chose to use only *G ruber* (w, pooled) Mg/Ca and δ^18^O_c_ data to calculate representative δ^18^O_sw_ values for each time interval, for use with both the *G. ruber* (w) and *T. sacculifer* (w/s) δ^18^O_c_ IFA datasets. Considering both planktonic species are considered as shallow-dwellers with similar living depths and an affinity for the DCM, the utilisation of common δ^18^O_sw_ values is applicable^13,28,30^.

The *G. ruber* Mg/Ca-temperature Eq. (1) from^55^ (temperature calibration range: ~ 22–27 °C), similarly applied in the Maldivian study of^28^, was used in this study:

1 $$Mg/Ca=0.34\left(\pm 0.08\right)\mathrm{exp}(0.102\left(\pm 0.010\right)*T)$$

The applied δ^18^O-temperature species-specific equations (Eqs. 2 and 3) were previously utilised in the local study by^28^. Both the *G. ruber* (Eq. 2) and *T. sacculifer* (Eq. 3) equations are from the Indian Ocean study of^59^ (temperature calibration range: ~ 20–31 °C):

2 $$T=12.75-5({\delta }^{18}{O}_{c}-{\delta }^{18}{O}_{sw})$$

3 $$T=11.95-5.26({\delta }^{18}{O}_{c}-{\delta }^{18}{O}_{sw})$$

Using the above equations, the range in temperature estimates are obtained as follows (Fig. 4):

1. The mean *G. ruber* (w) Mg/Ca measurements are used together with Eq. (1) to calculate a temperature estimate for each time point (Supplementary Table 1). Since the Mg/Ca calcification temperatures are based on 30 pooled specimens, they are considered to reflect mean calcification temperatures.
2. The Mg/Ca derived temperature estimates are then used together with the mean traditional (pooled) *G. ruber* (w) δ^18^O_c_ data and Eq. (2) to calculate representative δ^18^O_sw_ values for each time point (Supplementary Table 2). As these are calculated from pooled samples, they are considered to mirror mean δ^18^O_sw_ values for both the Recent and fossil populations.
3. The *G. ruber* (w) and *T. sacculifer* (w/s) IFA datasets are then used, together with the relevant species-specific δ^18^O-temperature equations and δ^18^O_sw_ values, to calculate the spread in temperature estimates (Fig. 4, Supplementary Tables 3–4).

*Trilobatus sacculifer* (w/s) data from the glacial maxima of MIS12 are included in the study to illustrate the applicability of the IFA method, however, as they do not contribute to the discussion on bleaching thresholds, they are discussed further in the Supplementary Materials (Supplementary Figs. 1, 3).

Finally, the temperature estimates for the shallow-dwelling symbiont-bearing benthic *A. lessonii* are obtained using the genus-specific δ^18^O-temperature equation of^60^ (Eq. 4) (Supplementary Tables 5–6).

4 $$T=16.3-4.24({\delta }^{18}{O}_{c}-{\delta }^{18}{O}_{sw})$$

Considering the benthic specimens were deemed living at the time of collection (Rose Bengal stained), a mean regional surface (0 m) δ^18^O_sw_ value (0.49 ‰) is used together with the δ^18^O_c_ data in the calculations (Supplementary Tables 5–6).

## Supplementary Information

Supplementary Information.

## Data Availability

All new raw IFA data has been made available on The World Data Center PANGAEA.
