# Supplementary material for: Using past interglacial temperature maxima to explore transgressions in modern Maldivian coral and Amphistegina bleaching thresholds
Source: Sci Rep. 2021 May 13;11:10267. doi: 10.1038/s41598-021-89697-0 (PMC8119970; doi:10.1038/s41598-021-89697-0)
Supplement: Supplementary file 1 — Supplementary Information. [file 41598_2021_89697_MOESM1_ESM.pdf]

Supplementary Material to:

Using past interglacial temperature maxima to explore transgressions in modern Maldivian coral and *Amphistegina* bleaching thresholds

Stephanie Stainbank[1*], Dick Kroon[2], Erica S. de Leau[2], Silvia Spezzaferri[1]

[1] University of Fribourg, Department of GeoSciences, Chemin du Musée 6, 1700 Fribourg, Switzerland

[2] University of Edinburgh, School of GeoSciences, Grant Institute, The King's Buildings, James Hutton Road, EH9 3FE Edinburgh, United Kingdom

*Corresponding author

E-mail: stephaniehayman.23@gmail.com

The establishment of seawater temperature estimates, using foraminiferal geochemistry, is not straight-forward, especially given the differing species-specific salinity and pH sensitivities, within shell geochemical composition variability in conjunction with the ever increasing number of published Mg/Ca- and δ^{18}O-temperature calibrations [1–6]. Specifically, [6] have shown the gametogenic calcite of *T. sacculifer*, one of the species used in this study, is significantly enriched in Mg (average of 230 %) in comparison to the ontogenetic calcite. Given the specimens used in this study, had a sac-like final chamber and thus are assumed to have undergone reproduction [7] it calls into question any traditional whole-shell (pooled) Mg/Ca measurements obtained from this species. Moreover, considering the Mg/Ca derived temperature estimates are used to calculate δ^{18}O seawater ($\delta^{18}O_{sw}$) values, which subsequently are used together with the $\delta^{18}O_c$ individual foraminiferal analysis (IFA) for temperature calculations, any biases will inherently be incorporated into all calculations. Based on these considerations and given both target foraminiferal species in this study (i.e., *G. ruber* (w) and *T. sacculifer* (w/s)) are shallow dwellers, we opted to exclusively use the *G. ruber* (w) traditional whole-shell data for calculation of $\delta^{18}O_{sw}$ estimates for all investigated time intervals (Supplementary Figs. 1-2).

Another complication is the possibility of bioturbation, which would result in the inherent reworking of specimens from different time points. Considering the target study intervals are interglacial δ^{18}O minima, reworking of samples from cooler glacials is a possibility and would bias the datasets towards higher δ^{18}O/cooler temperatures. While the IQR method would identify outliers in the datasets, it would not necessarily identify reworked glacial samples within the interglacial datasets. In this respect, the additional use of the Recent datasets to designate a rudimentary cut-off point for the highest $\delta^{18}O_c$ value to expect during past interglacial minima proved satisfactory. Supplementary Figure 2 shows the entire IFA datasets, including identified outliers and reworked samples. As can be seen, while the combination of these two methods does eliminate many of the higher $\delta^{18}O_c$ values, it does not significantly influence the overall interpretations. This is especially true when assessing the percentage of each dataset above the Maldivian coral bleaching threshold.

Lastly, samples from a glacial maxima (MIS12) were analysed and included as a comparison to illustrate the applicability of the applied IFA technique (Supplementary Figs. 1, 3). Due to sample availability, only *T. sacculifer* (w/s) IFA was included. However, in order to calculate a representative $\delta^{18}O_{sw}$ value, traditional (pooled) Mg/Ca and $\delta^{18}O_c$ measurements were

obtained for *G. ruber* (w) along with *T. sacculifer* (w/s). The *T. sacculifer* (w/s) data demonstrates the cohesion between the traditional pooled data (yellow triangles and circle) and the peak of the IFA dataset. Considering the included comparison is from a cold glacial maximum, the IQR method was used to identify outliers in the IFA dataset. Importantly, while the data is not expected to exceed the bleaching threshold, it still shows the traditional pooled data represents more a mean, whereas the benefit of the IFA technique allows the upper temperature extremes to be explored.

Supplementary Tables 1 – 6 summarise the foraminiferal geochemical data used in this study together with the $\delta^{18}O_{sw}$ and temperature calculations.

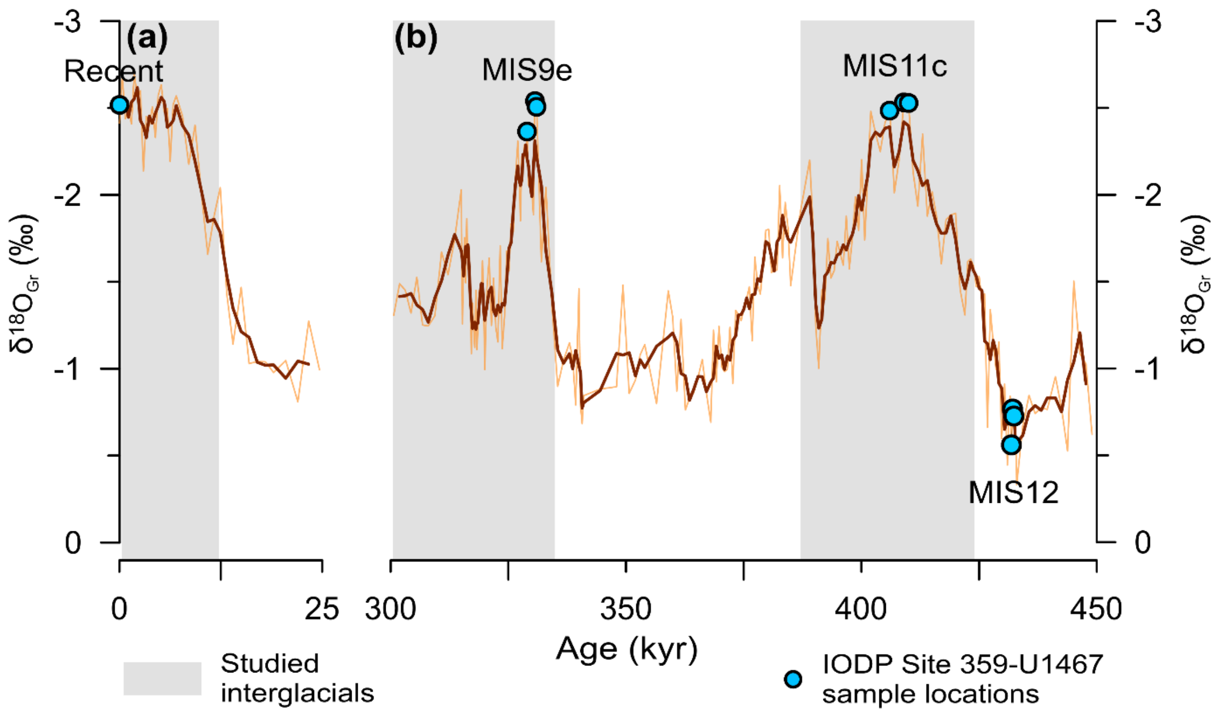

Supplementary Figure 1. Long-term IODP359 Site U1467 $\delta^{18}O_c$ *G. ruber* (w) records [9] **together with the exact down-core location of all samples used in this study (blue circles).** Thin orange line is the $\delta^{18}O_c$ data, with a 3-pt moving average smoothing applied to (thick line) to highlight the cycles. **(a)** Location of the Recent (Mudline) sample with **(b)** the location of the three MIS9e (U1467C, 2H6, 0-1 cm; U1467C, 2H6, 15-16 cm; U1467C, 2H6, 18-19 cm), MIS11c (U1467B, 3H2, 147-148 cm; U1467B, 3H3, 9-10 cm; U1467B, 3H3, 12-13 cm) and MIS12 (U1467B, 3H3, 102-103 cm; U1467B, 3H3, 105-106 cm; U1467B, 3H3, 108-109 cm) samples used. MIS = Marine Isotope Stages, with MIS1, MIS9 and MIS11 highlighted in grey shading.

Supplementary Figure 2. Full spread (including previously excluded outliers) in $\delta^{18}O_c$ individual foraminiferal analysis (IFA) data and seawater temperature estimates. Histograms of the full $\delta^{18}O_c$ IFA data spread (counts = number of measurements) and the corresponding temperatures (box and whisker plots) for **(a)** the Recent (blue), **(b)** MIS9e (green) and **(c)** MIS11c (grey) for both **(1)** *G. ruber* (w) and **(2)** *T. sacculifer* (w/s). The identified outliers for each dataset are shown in black. Traditional $\delta^{18}O_c$ values (based on pooled specimens) are shown (yellow triangles) for each sample together with the mean $\delta^{18}O$ derived temperature (based on pooled specimens) (yellow circles). Temperatures > the Maldives coral bleaching threshold (~30.9 °C [8]) are shown in red shading. Numbers indicate the percentage of the IFA temperature estimates, from each dataset, > this bleaching threshold. Note: MIS9e and MIS11c represent the pooled data from three samples.

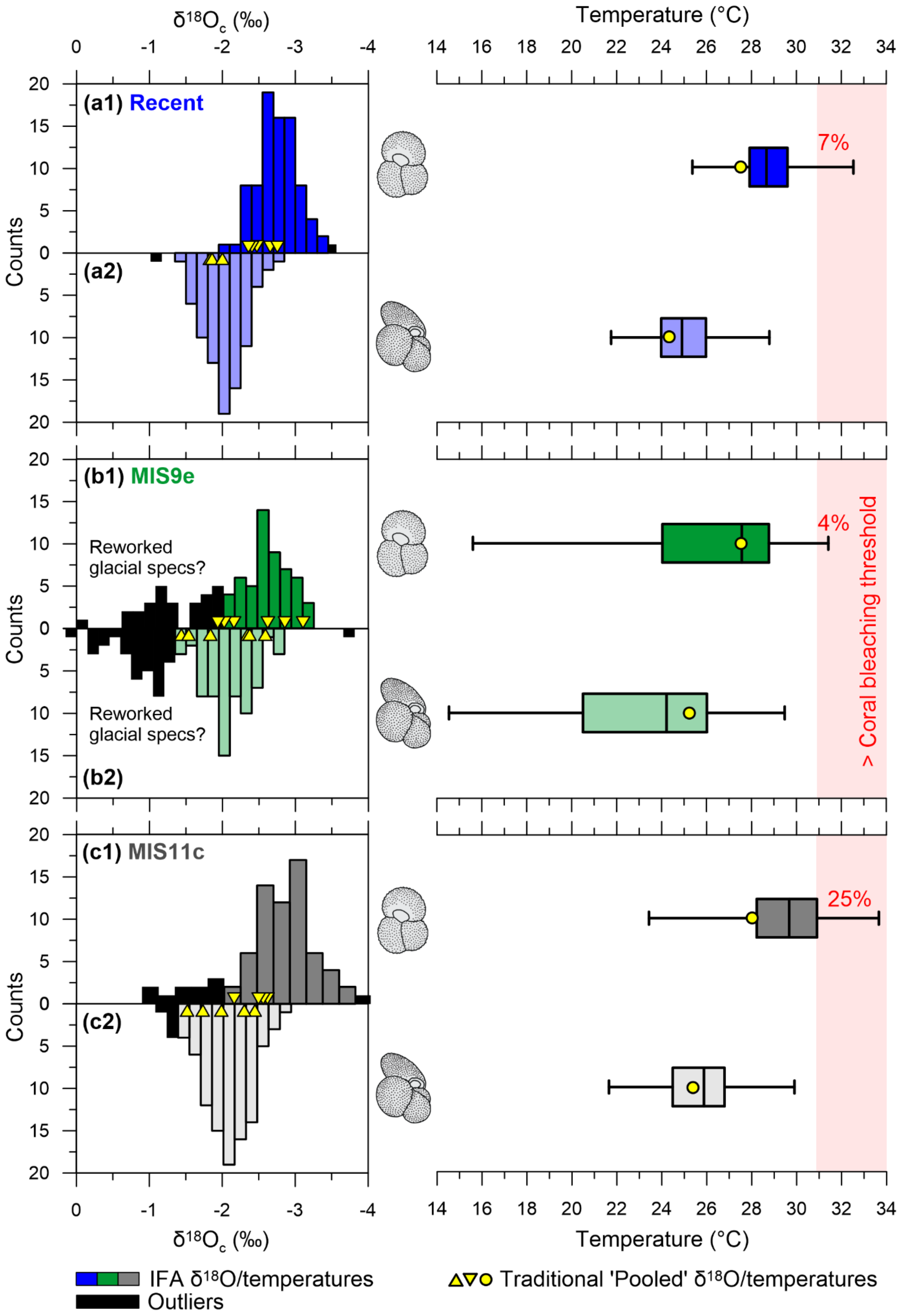

δ18Oc (‰)
Temperature (°C)
Counts
(a1) Recent
(a2)
7%
(b1) MIS9e
Reworked glacial specs?
Reworked glacial specs?
(b2)
4%
> Coral bleaching threshold
(c1) MIS11c
(c2)
25%
IFA δ18O/temperatures
Outliers
Traditional 'Pooled' δ18O/temperatures

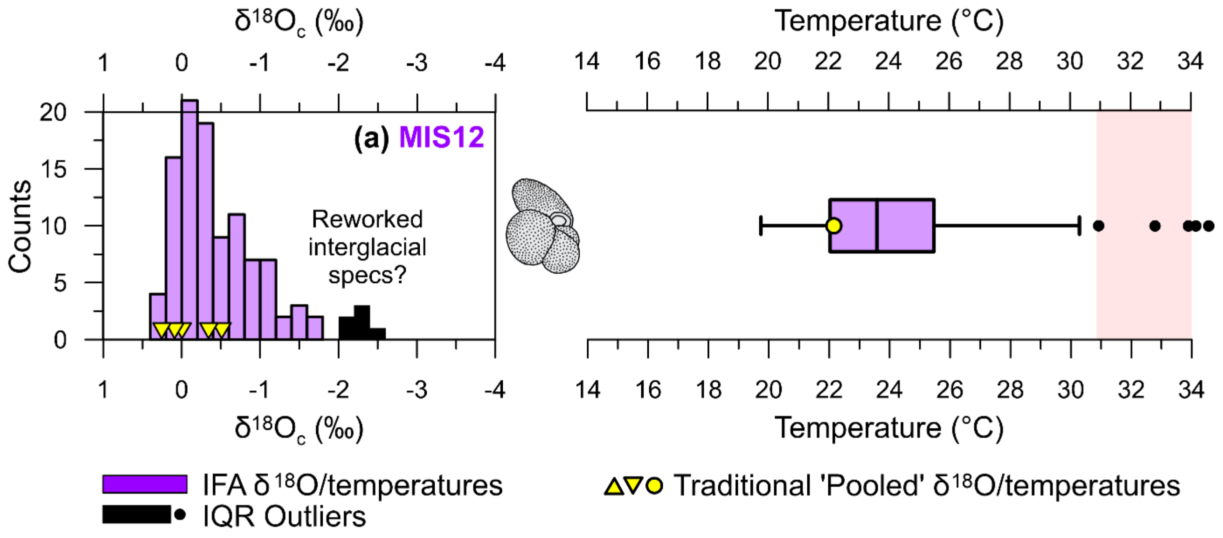

Supplementary Figure 3. Spread in δ^{18}O$_c$ individual foraminiferal analysis (IFA) data and seawater temperature estimates for MIS12. Histograms of the δ^{18}O$_c$ IFA data spread (counts = number of measurements) and the corresponding temperatures (box and whisker plots) for **(a)** MIS12 *T. sacculifer* (w/s). The IQR identified outliers are shown in black. Traditional δ^{18}O$_c$ values (based on pooled specimens) are shown (yellow triangles) together with the mean δ^{18}O$_c$ derived temperature (based on pooled specimens) (yellow circle). Temperatures > the Maldives coral bleaching threshold (~30.9 °C [8]) are shown in red shading. Note: MIS12 represents the pooled data from three samples.

Supplementary Table 1. Traditional (pooled) whole-shell geochemical data from Site IODP359-U1467. w = white, w/s = with/sac. Note: the symbol * indicates data used from [10], † indicates data from [11], ‡ indicates data from [9] and ** indicates data from [12].

Sample		*G. ruber* (w)		*T. sacculifer* (w/s)
		$\delta^{18}O$ (‰)	Mg/Ca (mmol/mol)	$\delta^{18}O$ (‰)
Mudline	A, Mudline	-2.47†	5.66†	-2.03†
	A, Mudline	-2.40†	5.65†	-1.87†
	A, Mudline	-2.35†	-	-
	A, Mudline	-2.66†	-	-
	A, Mudline	-2.75†	-	-
	B, Mudline	-2.47†	--	-1.83†
MIS9e	C2H6, 0-1 cm	-3.10	5.62‡	-2.60
	C2H6, 0-1 cm	-2.16	-	-1.46
	C2H6, 15-16 cm	-2.85	5.75‡	-1.46
	C2H6, 15-16 cm	-2.01	-	-2.35
	C2H6, 18-19 cm	-2.62	5.65‡	-1.84
	C2H6, 18-19 cm	-2.05	-	-2.39
MIS11c	B3H2, 147-148 cm	-2.48	5.78‡	-2.45
	B3H2, 147-148 cm	-2.48‡	-	-2.10
	B3H3, 9-10 cm	-2.53*	6.31**	-1.98**
	B3H3, 9-10 cm	-2.60**	5.44**	-2.30**
	B3H3, 9-10 cm	-	5.97‡	
	B3H3, 12-13 cm	-2.53*	5.87**	-1.51**
	B3H3, 12-13 cm	-2.23**	5.45**	-1.73**
	B3H3, 12-13 cm	-	5.88‡	
MIS12	B3H3, 102-103 cm	-0.56*	4.99**	-0.36**
	B3H3, 102-103 cm	-0.90**	-	0.08**
	B3H3, 105-106 cm	-0.77*	5.11**	0.25**
	B3H3, 105-106 cm	-1.11**	4.87‡	0.08**
	B3H3, 108-109 cm	-0.73*	4.80**	0.00**
	B3H3, 108-109 cm	-0.90**	4.96‡	-0.52**

Supplementary Table 2. Mean $\delta^{18}O_{sw}$ estimates obtained for each time interval using the mean *G. ruber* (w) Mg/Ca derived temperatures together with the traditional 'pooled' $\delta^{18}O$ data. *Corrected for an ice-volume effect of -1.1‰.

**Sample**	**$\delta^{18}O_{sw}$**
Mudline	0.66
MIS9e	0.73
MIS11c	0.76
MIS12	0.99*

Supplementary Table 3. Overview of the entire $\delta^{18}O_c$ Individual Foraminifera Analyses (IFA) datasets for each sample and planktonic species. SE = standard error, SD = standard deviation, w = white, w/s = with/sac.

**Sample**	**$\delta^{18}O_c$ IFA**	**#**	**Mean**	**SE of the mean**	**SD**	***p* Value Shapiro**	**Skewness**
Mudline	*G. ruber* (w)	84	-2.76	0.03	0.28	0.98	-0.18
	T. sacculifer (w/s)	81	-2.03	0.03	0.29	0.83	0.07
MIS9e	*G. ruber* (w)	83	-2.19	0.08	0.72	0.00*	0.83
	T. sacculifer (w/s)	103	-1.67	0.07	0.69	0.02*	0.25
MIS11c	*G. ruber* (w)	74	-2.70	0.07	0.59	0.00*	0.85
	T. sacculifer (w/s)	100	-2.06	0.03	0.35	0.80	0.25
MIS12	*T. sacculifer* (w/s)	107	-0.52	0.06	0.65	0.00*	-1.24

*null hypothesis is rejected = dataset is not normally distributed

Supplementary Table 4. Temperature estimates for the Individual Foraminifera Analyses (IFA) (excluding identified outliers) together with the mean values (± SD) based on pooled specimens (bold). w = white, w/s = with/sac. *indicates IFA min/max if outliers were to be included.

Sample	Species	Temperatures (°C)
Mudline	*G. ruber* (w) IFA min	25.37 °C
	G. ruber (w) IFA max	31.84 °C / *32.54 °C
	G. ruber **(w) δ^{18}O (pooled) mean**	**27.56 ± 0.70 °C**
	T. sacculifer (w/s) IFA min	21.75 °C/ *20.17 °C
	T. sacculifer (w/s) IFA max	28.79 °C
	T. sacculifer **δ^{18}O (w/s) (pooled) mean**	**24.33 ± 0.45 °C**
MIS9e	*G. ruber* (w) IFA min	25.72 / *15.60 °C
	G. ruber (w) IFA max	31.41 °C
	G. ruber **(w) δ^{18}O (pooled) mean**	**27.59 ± 2.08 °C**
	T. sacculifer (w/s) IFA min	22.10 °C / *14.54 °C
	T. sacculifer (w/s) IFA max	29.48 °C / *34.38 °C
	T. sacculifer **δ^{18}O (w/s) (pooled) mean**	**25.21 ± 2.40 °C**
MIS11c	*G. ruber* (w) IFA min	25.94 °C/ *20.17 °C
	G. ruber (w) IFA max	33.66 °C / *35.36 °C
	G. ruber **(w) δ^{18}O (pooled) mean**	**27.82 ± 0.64 °C**
	T. sacculifer (w/s) IFA min	22.40 °C/ *20.72 °C
	T. sacculifer (w/s) IFA max	29.90 °C
	T. sacculifer **δ^{18}O (w/s) (pooled) mean**	**25.38 ± 1.39 °C**
MIS12	*T. sacculifer* (w/s) IFA min	19.74 °C
	T. sacculifer (w/s) IFA max	30.28 °C / *35.33 °C
	T. sacculifer **δ^{18}O (w/s) (pooled) mean**	**22.20 ± 1.42 °C**

Supplementary Table 5. Modern $\delta^{18}O_{sw}$ values for the regional surface (0 m) waters.

**Reference**	**Longitude**	**Latitude**	**Month**	**Season**	**$\delta^{18}O_{sw}$ (‰)**
[13]	73.82 °E	0.10 °N	June	Summer	0.38
[14]	76.40 °E	2.00 °N	June	Summer	0.32
[15]	68.38 °E	8.03 °N	January	Winter	0.51
[15]	67.84 °E	7.01 °N	January	Winter	0.74
Average					0.49

Supplementary Table 6. Stable isotopic measurements of single, Rose-Bengal stained *A. lessonii* specimens from the Maldives. All specimens were collected from 10 m water depth during the 2015 IUCN REGENERATE cruise [16].

Sample Name	Stable Isotopes		T (°C)
	$\delta^{13}C_c$	$\delta^{18}O_c$	
Maya1.1	-0.23	-3.07	30.24
Maya1.1	-0.25	-3.27	31.11
Maya1.1	-0.09	-2.93	29.64
Maya3.1	0.06	-2.90	29.52
Maya3.1	-0.85	-2.23	26.70
Ras2.2	-0.70	-3.18	30.70

References

1. Anand, P., Elderfield, H. & Conte, M. H. Calibration of Mg/Ca thermometry in planktonic foraminifera from a sediment trap time series. *Paleoceanography* **18**, 1050 (2003).
2. Mulitza, S. *et al.* Temperature: $\delta^{18}O$ relationships of planktonic foraminifera collected from surface waters. *Palaeogeogr. Palaeoclimatol. Palaeoecol.* **202**, 143–152 (2003).
3. Regenberg, M., Steph, S., Nürnberg, D., Tiedemann, R. & Garbe-Schönberg, D. Calibrating Mg/Ca ratios of multiple planktonic foraminiferal species with $\delta^{18}O$-calcification temperatures: Paleothermometry for the upper water column. *Earth Planet. Sci. Lett.* **278**, 324–336 (2009).
4. Gray, W. R. & Evans, D. Nonthermal influences on Mg/Ca in planktonic foraminifera: A review of culture studies and application to the Last Glacial Maximum. *Paleoceanogr. Paleoclimatology* **34**, 306–315 (2019).
5. Elderfield, H., Vautravers, M. & Cooper, M. The relationship between shell size and Mg/Ca, Sr/Ca, $\delta^{18}O$, and $\delta^{13}C$ of species of planktonic foraminifera. *Geochemistry, Geophys. Geosystems* **3**, (2002).
6. Nürnberg, D., Bijma, J. & Hemleben, C. Assessing the reliability of magnesium in foraminiferal calcite as a proxy for water mass temperatures. *Geochim. Cosmochim. Acta* **60**, 803–814 (1996).
7. Schiebel, R. & Hemleben, C. *Planktic Foraminifers in the Modern Ocean*. (Springer-Verlag, 2017).
8. Perry, C. T. & Morgan, K. M. Bleaching drives collapse in reef carbonate budgets and reef growth potential on southern Maldives reefs. *Sci. Rep.* **7**, 1–9 (2017).
9. Stainbank, S. *et al.* Assessing the impact of diagenesis on foraminiferal geochemistry from a low latitude, shallow-water drift deposit. *Earth Planet. Sci. Lett.* 116390 (2020).
10. Stainbank, S., Spezzaferri, S., Kroon, D., de Leau, E. S. & Rüggeberg, A. The Planktonic foraminifera *Globigerinoides eoconglobatus* n. sp. in a glacial–interglacial context: IODP359 Sites U1467 and U1468. *Swiss J. Geosci.* **111**, 483–494 (2018).
11. Stainbank, S. *et al.* Controls on planktonic foraminifera apparent calcification depths for the northern equatorial Indian Ocean. *PLoS One* **14**, e0222299 (2019).
12. Stainbank, S. *et al.* Monsoon and tropical climate forcing on the physicochemical and thermocline characteristics of the Maldives Inner Sea: Insights from Marine Isotope Stages 1–2 and 10–13. *Paleoceanogr. Paleoclimatology* (accepted).
13. Duplessy, J. C., Bé, A. W. H. & Blanc, P. L. Oxygen and carbon isotopic composition and biogeographic distribution of planktonic foraminifera in the Indian Ocean. *Palaeogeogr. Palaeoclimatol. Palaeoecol.* **33**, 9–46 (1981).
14. Dahl, K. A. & Oppo, D. W. Sea surface temperature pattern reconstructions in the Arabian Sea. *Paleoceanography* **21**, PA1014 (2006).

15. Srivastava, R., Ramesh, R., Prakash, S., Anilkumar, N. & Sudhakar, M. Oxygen isotope and salinity variations in the Indian sector of the Southern Ocean. *Geophys. Res. Lett.* **34**, L24603 (2007).
16. Pisapia, C. *et al. Baseline assessment of coral reefs of North Ari Atoll, Maldives*. *Gland, Switzerland: IUCN and Government of Maldives* https://portals.iucn.org/library/sites/library/files/documents/2017-017_0.pdf (2017).
